# Transcription factor NF-κB promotes acute lung injury *via* microRNA-99b-mediated PRDM1 down-regulation

**DOI:** 10.1074/jbc.RA120.014861

**Published:** 2021-01-13

**Authors:** Jie Zhao, Fei Xie, Ruidong Chen, Zhen Zhang, Rujun Dai, Na Zhao, Rongxin Wang, Yanhong Sun, Yue Chen

**Affiliations:** 1 The Second Department of Pediatric, Cangzhou Central Hospital, Cangzhou, P. R. China; 2 The Six Department of Pediatric, Cangzhou Central Hospital, Cangzhou, P. R. China

**Keywords:** NF-κB(p65, ), microRNA-99b, PRDM1, inflammatory factors, acute lung injury, lung, lung injury, cell biology, cell death, cell cycle, NF-kB(p65)

## Abstract

Acute lung injury (ALI), is a rapidly progressing heterogenous pulmonary disorder that possesses a high risk of mortality. Accumulating evidence has implicated the activation of the p65 subunit of NF-κB [NF-κB(p65)] activation in the pathological process of ALI. microRNAs (miRNAs), a group of small RNA molecules, have emerged as major governors due to their post-transcriptional regulation of gene expression in a wide array of pathological processes, including ALI. The dysregulation of miRNAs and NF-κB activation has been implicated in human diseases. In the current study, we set out to decipher the convergence of miR-99b and p65 NF-κB activation in ALI pathology. We measured the release of pro-inflammatory cytokines (IL-1β, IL-6, and TNFα) in bronchoalveolar lavage fluid using ELISA. MH-S cells were cultured and their viability were detected with cell counting kit 8 (CCK8) assays. The results showed that miR-99b was up-regulated, while PRDM1 was down-regulated in a lipopolysaccharide (LPS)-induced murine model of ALI. Mechanistic investigations showed that NF-κB(p65) was enriched at the miR-99b promoter region, and further promoted its transcriptional activity. Furthermore, miR-99b targeted PRDM1 by binding to its 3'UTR, causing its down-regulation. This in-creased lung injury, as evidenced by increased wet/dry ratio of mouse lung, myeloperoxidase activity and pro-inflammatory cytokine secretion, and enhanced infiltration of inflammatory cells in lung tissues. Together, our findings indicate that NF-κB(p65) promotion of miR-99b can aggravate ALI in mice by down-regulating the expression of PRDM1.

Acute lung injury (ALI) is a prevalent disease with exceedingly-high rates of morbidity and mortality. ALI can often predispose patients to acute respiratory distress syndrome (ARDS) (1), which results from an acute injury such as sepsis, aspiration, shock, pneumonia (2). ALI is triggered on excessive neutrophil infiltration into the lung tissues and lung endothelium and epithelium thus resulting in edema along with gas exchange deterioration (3). Lipopolysaccharide (LPS) has been commonly used to induce ALI in a murine model (4, 5). Numerous studies have even reported that LPS can induce the expressions of inflammatory mediators activating the nuclear factor-kappaB (NF-κB) signaling pathway, causing the formation of inflammasomes (6, 7, 8). Inflammasome activation stimulates cysteine protease caspase-1 that has capability of cleaving the precursor forms of pro-inflammatory cytokines, such as interleukin (IL)-1β and IL-18 (9, 10, 11). Once the organs fail to burden much inflammasome activity, the human body elicits a severe immune response that stimulates excessive release of pro-inflammatory cytokines, thus prompting some inflammatory diseases including ALI (12, 13).

MicroRNAs (miRNAs), 21–23 nucleotides in length, possess the ability to negatively regulate gene expression by repression of mRNA translation repression or promotion of mRNA degradation (14, 15, 16). Mounting evidence further supports that abnormal expression of miRNAs is associated with some of inflammatory lung diseases. For instance, up-regulated levels of miR-125b are known to reduce LPS-induced pulmonary inflammation in mice (17). In addition, LPS-induced inflammatory response was limited on miR-212-3p overexpression in murine macrophages (18). More notably, a previous study documented several miRNA candidates with altered expressions in ALI. miR-99b shows a positive-correlation with the activation of NF-κB (19), which regulates the expressions of a large variety of genes that are involved in numerous processes like inflammatory and immune responses of the cell, cell growth, and development (20). Moreover, transcriptional repressor PR (PRDI-BF1-RIZ) domain zinc finger protein 1 (PRDM1) has also been identified to be a downstream effector of the NF-κB (21). PRDM1 (also referred to as Blimp-1) was originally identified as a post-inductive silencer of interferon beta (IFN-β) gene expression and controls cell fate decisions in multiple tissue contexts (22). However, the mechanism underlying the role of miR-99b and NF-κB in ALI remains unclear. In the current study, we performed experiments using ALI mouse models, and found miR-99b expression was increased in the lung tissues. In addition, we observed that NF-κB could increase miR-99b expression and deteriorate ALI of mice induced by LPS. As a result, we speculated whether the miR-99b/PRDM1 axis regulated the development of ALI. Therefore, we set out to elucidate the mechanism by which miR-99b affects the processes of ALI by means of LPS-induced MH-S cells and a murine model of LPS-induced ALI.

## Results

### miR-99b is highly-expressed in ALI mouse models

**Figure 1:**
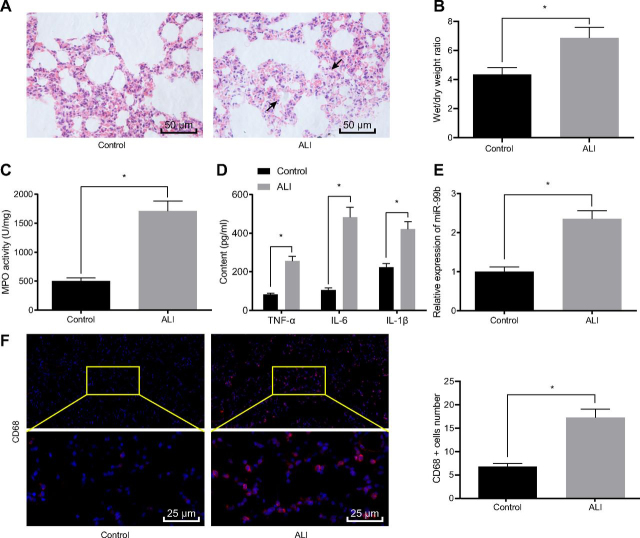
**High expression of miR-99b is observed in ALI mice.***A*, Detection of pathological changes in lung tissues of control and ALI mice by HE staining (The arrow indicates the area of lung injury). *B*, W/D ratio in lung tissues of control and ALI mice. *C*, MPO activity in lung tissues of control and ALI mice. *D*, Detection of inflammatory factors TNFα, IL-6, and IL-1β expression in the BALF of control and ALI mice by ELISA. *E*, miR-99b expression in lung tissues of control and ALI mice detected by RT-qPCR. *F*, Detection of CD-68 antibody expression in lung tissues of control and ALI mice by immunofluorescence. The measurement data were presented as mean ± standard deviation. The data of two groups were analyzed by independent sample *t test*. * indicated *p* < 0.05, *n* = 8.

Firstl the results of hematoxylin-eosin (HE) staining illustrated disordered alveolar structure, thickened alveolar wall, obvious alveolar septum, interstitial edema and inflammatory cell infiltration in the lung tissues of mice in the ALI group compared with the control group (Fig. 1*A*). The dry/wet weight (W/D) ratio of the mice in the ALI group was elevated relative to the control group (Fig. 1*B*). Myeloperoxidase (MPO) activity was elevated in mice after ALI treatment (Fig. 1*C*). ELISA (ELISA) was further employed to detect the expression patterns of inflammatory factor tumor necrosis factor-α (TNFα), IL-6, and IL-1β in the BALF, and the results showed that LPS treatment led to elevated TNFα, IL-6, and IL-1β in the BALF of mice (Fig. 1*D*).

Subsequently, the expression patterns of miR-99b in lung tissues were detected with reverse transcription quantitative PCR (RT-qPCR), and the results showed a significant elevation in miR-99b expression levels in the lung tissues of mice of the ALI group compared with the control group (Fig. 1*E*). Additionally, immunofluorescence staining analysis revealed a significant increase in the number of macrophages in the lung tissues of mice from the ALI group when compared with the control group (Fig. 1*F*). These findings indicated that miR-99b was highly-expressed in mice with ALI.

### Silencing of miR-99b protects mice against ALI

**Figure 2:**
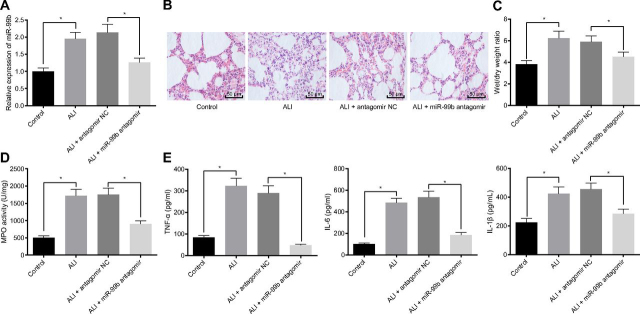
**Silencing miR-99b relieves lung injury in ALI mice.** Control mice served as the control and ALI mice were treated with miR-99b antagomir. *A*, Detection of miR-99b expression in mouse lung tissues by RT-qPCR. *B*, Detection of pathological changes of mouse lung tissues by HE staining. *C*, W/D ratio in lung tissues of mice. *D*, MPO activity in lung tissues of mice; *E*, Detection of inflammatory factors TNFα, IL-6, and IL-1β expression in the BALF of mice by ELISA. The measurement data were presented as mean ± standard deviation. The data of two groups were analyzed by independent sample *t test*. * indicated *p* < 0.05, *n* = 8.

To further explore the involvement of miR-99b in regulating ALI, the status of lung injury in mice was examined by treating ALI mice with miR-99b antagomir. RT-qPCR results revealed a significant decrease in the expression levels of miR-99b in the lung tissues of mice in the ALI + miR-99b antagomir group relative to the ALI + antagomir negative control (NC) group (Fig. 2*A*), indicating successful silencing of miR-99b in ALI mice. Meanwhile, HE staining demonstrated that inflammatory cell infiltration was reduced in the lung tissues of mice in the ALI + miR-99b antagomir group compared with the ALI + antagomir NC group, in addition to marked improvements in alveolar septum and pulmonary interstitial edema (Fig. 2*B*). In addition, decreased lung W/D ratio (Fig. 2*C*), MPO activity (Fig. 2*D*), and TNFα, IL-6, and IL-1β expression levels were observed in the BALF (Fig. 2*E*) in mice in the ALI + miR-99b antagomir group compared with the ALI + antagomir NC group. Taken together, these findings indicate that silencing miR-99b could relieve lung injury in ALI mice.

### NF-κB(p65) in macrophages promotes miR-99b transcription and cell injury

**Figure 3:**
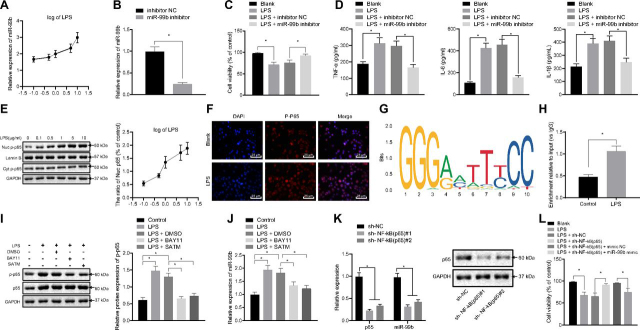
**NF-κB(p65) enhances miR-99b transcription to promote the inflammatory damage of cells.***A*, miR-99b expression was detected by RT-qPCR in cells after LPS treatment at different concentrations. *B*, Expression of miR-99b in cells was detected by RT-qPCR after silencing miR-99b. *C*, The effects of silencing miR-99b on macrophage viability were detected by CCK8 assay. *D*, Detection of inflammatory factor TNFα, IL-6, and IL-1β expression in macrophage culture supernatant by ELISA. *E*, Western-blot was applied to detect the translocation of NF-κB(p65) into nuclei in cells. *F*, Effects of LPS treatment on p-p65 expression was detected by immunofluorescence. *G*, Binding site of NF-κB(p65) in the miR-99b promoter region. *H*, The enrichment of NF-κB(p65) in the miR-99b promoter region of MH-S cells was detected by ChIP test. *I*, p-p65 and p65 expression was detected by western-blot in MH-S cells after treatment with NF-κB inhibitor. *J*, The expression of miR-99b was detected by RT-qPCR in MH-S cells after treatment with NF-κB inhibitor. *K*, Expression of p65 and miR-99b was detected by western-blot and RT-qPCR in MH-S cells after silencing p65. *L*, Cell viability changes were detected by CCK8 test upon treatment with sh-NF-κB(p65) or in combination with miR-99b mimic. The measurement data were presented as mean ± standard deviation. The data of two groups were analyzed by independent sample *t test*. The multi-group comparison was analyzed by one-way ANOVA with Tukey's post-hoc test. * indicated *p* < 0.05. The experiment was repeated three times.

Additionally, MH-S cell models of ALI were simulated by LPS treatment to further study the mechanism of miR-99b in ALI regulation. RT-qPCR was then applied to detect the expression patterns of miR-99b in the cells following treatment with different concentrations of LPS, and the results showed that miR-99b expression was elevated in the cells following escalating concentration of LPS treatment (Fig. 3*A*).

Subsequently, miR-99b was silenced in the LPS-stimulated cells, and RT-qPCR results revealed a decrease in miR-99b expression upon miR-99b inhibitor introduction (Fig. 3*B*). The results of cell counting kit 8 (CCK8) and ELISA experiments showed that LPS treatment repressed MH-S cell viability and enhanced TNFα, IL-6 and IL-1β levels. However, compared with the LPS + inhibitor NC group, cell viability was promoted, whereas the expression levels of TNFα, IL-6 and IL-1β were all decreased in the LPS + miR-99b inhibitor group (Fig. 3*C*, *D*).

The expression levels of NF-κB(p-p65) in the cells were detected using nucleocytoplasmic separation experimentation, and it was found that the expression of NF-κB(p-p65) in the nucleus was markedly elevated with the increase of LPS concentration (Fig. 3*E*). Immunofluorescence was then employed to detect the treatment of LPS on p-p65 expression levels, and the results indicated that LPS treatment promoted NF-κB(p-p65) nucleation (Fig. 3*F*).

The TRANSFAC database was retrieved to predict the NF-κB(p65) binding sites with the miR-99b promoter region (Fig. 3*G*). Results of ChIP (ChIP) assay revealed a promoted NF-κB(p65) enrichment at the miR-99b promoter region following LPS treatment (Fig. 3*H*).

NF-κB inhibitor (10 μm BAY11-7082 and 100 μm SATM) was used to treat MH-S macrophages, and the expression levels of NF-κB(p-p65) were detected by Western-blot. The results showed a significant decrease in NF-κB(p65) and NF-κB(p-p65) expression levels (Fig. 3*I*). RT-qPCR was then applied to detect the miR-99b expression patterns, and it was found that miR-99b expression was inhibited in the presence of NF-κB inhibitor (Fig. 3*J*). Macrophages p65 was further silenced by shRNA transfection, and the expression levels of p65 and miR-99b were detected using Western-blot and RT-qPCR. The results illustrated a decline in p65 and miR-99b expression once NF-κB was silenced (Fig. 3*K*). Cell viability of LPS-stimulated MH-S was observed to be promoted upon NF-κB knockdown, while concurrent transfection of miR-99b mimic inhibited the viability (Fig. 3*L*). The above results suggested that NF-κB(p65) was enriched at the miR-99b promoter region, and promoted its transcriptional activity, thus accelerating inflammatory damage to the cells.

### miR-99b targets PRDM1 to down-regulate its expression and promote LPS-induced macrophage injury

**Figure 4:**
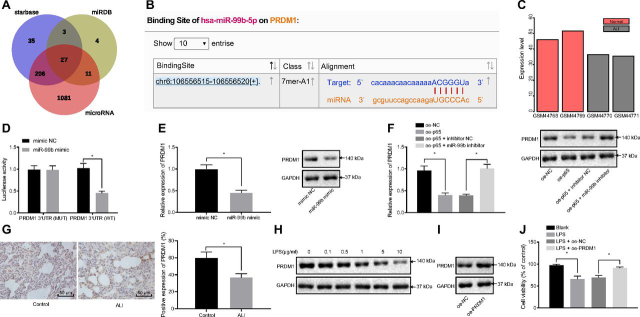
**miR-99b targetes PRDM1 to enhance LPS induced cell injury.** A: Enrichment analysis in the potential targeted genes of miR-99b by starBase, miRDB and microRNA databases. B: Binding sites of miR-99b in the 3'UTR of PRDM1 mRNA. C: The expression of PRDM1 in lung injury samples of the ALI-associated GSE2368 data set. D: Binding relationship between miR-99b and PRDM1 was detected by dual-luciferase reporter gene assay in HEK293 cells. E: PRDM1 expression in macrophages transfected with miR-99b mimic was detected by RT-qPCR and western-blot. F: PRDM1 expression in macrophages transfected with oe-p65 or in combination with miR-99b inhibitor was detected by RT-qPCR and western-blot. G: Immunohistochemistry was used to detect the positive expression of PRDM1 protein in lung tissues of ALI mice. H: Expression of PRDM1 in cells was detected by western-blot after treatment with different concentrations of LPS. I: Expression of PRDM1 in PRDM1-overexpressed cells was detected by western-blot. J: The effect of PRDM1 overexpression on LPS induced cell viability was detected by CCK8. The measurement data were presented as mean ± standard deviation. The data of two groups were analyzed by independent sample *t test*. The multi-group comparison was analyzed by one-way ANOVA with Tukey's post-hoc test. * indicated *p* < 0.05. The experiment was repeated three times.

The downstream target genes of miR-99b were predicted using starBase (http://starbase.sysu.edu.cn/), miRDB (http://mirdb.org/index.html) and microRNA databases (http://www.microrna.org/microrna/getMirnaForm.do). Following diagram analysis of the predicted miRNAs (Fig. 4*A*), 27 mRNAs were found at the intersection. Subsequently, ALI mRNA expression data set GSE2368 was obtained from the Gene Expression Omnibus (GEO) database (https://www.ncbi.nlm.nih.gov/geo/), which comprised of 2 normal samples and 2 lung injury samples, and a set of three candidate mRNAs were yielded. PRDM1 was the only overlapping gene with the predicted 27 mRNAs in the aforementioned three databases. Meanwhile, previous literature has shown that deletion of PRDM1 (Blimp1) in T cells decreased the expression levels of Foxp3 and CTLA-4, while increasing those of proinflammatory cytokines and the production of autoantibodies, including the increase of IgE (23). Therefore, it was hypothesized that the PRDM1 gene was involved in the regulation of ALI, and thus, was selected for further experimentation. The results further revealed that miR-99b-5p targeted the PRDM1 gene (Fig. 4*B*), and the PRDM1 expression in the GSE2368 data set was much lower in the lung injury samples relative to normal samples (Fig. 4*C*).

The results of dual-luciferase reporter gene assay demonstrated that over-expression of miR-99b inhibited the luciferase activity of WT (WT)-PRDM1-3'UTR, while exerting no effects on the luciferase activity of mutant (Mut)-PRDM1-3'UTR in HEK293 cells (Fig. 4*D*). The results of RT-qPCR and Western-blot showed that the mRNA and protein expression levels of PRDM1 were inhibited in miR-99b over-expressed MH-S cells (Fig. 4*E*). PRDM1 expression patterns were also detected in MH-S cells following p65 over-expression or in combination with miR-99b inhibition, the results of which showed that the PRDM1 levels were inhibited by over-expression (oe)-p65 compared with the oe-NC, and compared with oe-p65 + inhibitor NC, the PRDM1 levels were restored by oe-p65 + miR-99b inhibition (Fig. 4*F*). These further demonstrated that the PRDM1 gene may be involved in the regulation of lung injury *via* p65/miR-99b expression levels.

Subsequently, immunohistochemical detection was performed to examine the PRDM1 expression patterns in ALI mouse lung tissues, and a significant decrease in the PRDM1 expression was observed compared with the control group (Fig. 4*G*). Western-blot results showed a concentration-dependent decline of PRDM1 expression in MH-S cells after LPS stimulation (Fig. 4*H*). PRDM1 over-expression was then achieved by lentivirus (Fig. 4*I*). CCK8 assay revealed PRDM1 overexpression increased cell viability of MH-S cells after LPS stimulation (Fig. 4*J*). Collectively, these findings revealed that miR-99b targeted PRDM1 and inhibited its expression to increase the severity of LPS induced cell injury.

### PRDM1 over-expression inhibits ALI in mice

**Figure 5:**
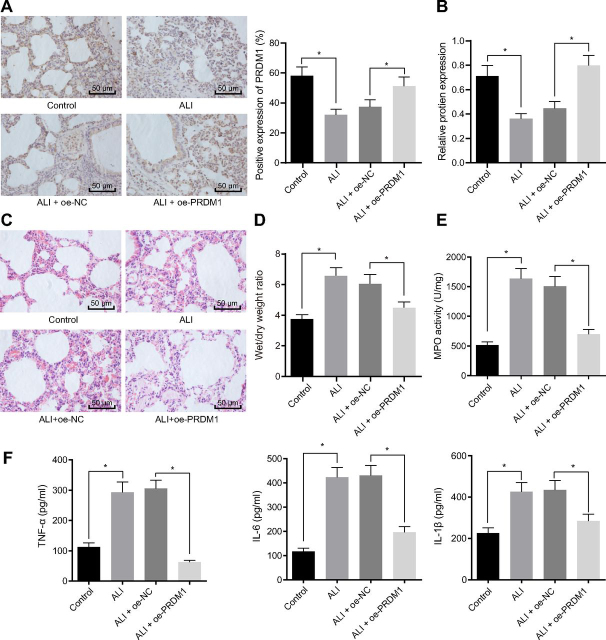
**PRDM1 overexpression inhibits lung injury in ALI mice.** A: Immunohistochemistry was used to detect the positive expression of PRDM1 protein in lung tissues of mice treated with oe-PRDM1. B: The expression of PRDM1 in lung tissues of mice treated with oe-PRDM1 was detected by western-blot. C: Pathological changes of lung tissues of mice treated with oe-PRDM1 were detected by HE staining. D: W/D ratio in lung tissues of mice treated with oe-PRDM1. E: MPO activity in lung tissues of mice treated with oe-PRDM1. F: Inflammatory factors TNFα, IL-6, and IL-1β expression in the BALF of mice treated with oe-PRDM1 was detected by ELISA. The measurement data were presented as mean ± standard deviation. The data of two groups were analyzed by independent sample *t test*. The one-way ANOVA was used for the comparison between multiple groups with Tukey's post-hoc test. * indicated *p* < 0.05, *n* = 8.

To further verify whether PRDM1 regulates ALI in mice, the extent of lung injury was examined by injecting lentivirus expressing oe-PRDM1 into ALI mice. The expression patterns of PRDM1 in lung tissues were first examined by immunohistochemistry after 28 days of injection. ALI mice presented an increase in PRDM1 expression in the lung tissues following infection of lentivirus expressing oe-PRDM1 (Fig. 5*A*). Western-blot yielded similar results regarding the protein expressions of PRDM1 to that of immunohistochemistry (Fig. 5*B*). HE staining was further applied to examine lung tissue damage, and it was observed that inflammatory cell infiltration and interstitial edema were decreased in mice of the ALI + oe-PRDM1 group compared with the ALI + oe-NC group (Fig. 5*C*). Moreover, the ALI + oe-PRDM1 group demonstrated decreased ratio of lung W/D (Fig. 5*D*), decreased MPO activity (Fig. 5*E*), and expression levels of inflammatory factor TNFα, IL-6, and IL-1β in BALF (Fig. 5*F*) compared with the ALI + oe-NC group. In a word, PRDM1 over-expression inhibited lung injury in ALI mice.

### NF-κB(p65) promotes ALI in mice through up-regulation of miR-99b by down-regulating PRDM1

**Figure 6:**
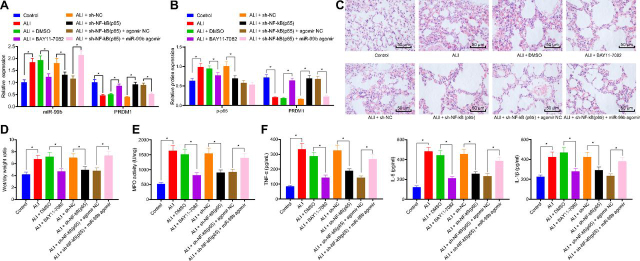
**NF-κB(p65) facilitates progression of ALI in mice by upregulating miR-99b and down-regulating PRDM1.** A: miR-99b expression and PRDM1 mRNA levels in lung tissues of ALI mice treated with BAY11-7082, sh-NF-κB(p65) or in combination with miR-99b agomir were detected by RT-qPCR. B: NF-κB(p65) and PRDM1 expression in lung tissues of mice treated with BAY11-7082, sh-NF-κB(p65) or in combination with miR-99b agomir was detected by western-blot. C: Pathological changes of lung tissues of ALI mice treated with BAY11-7082, sh-NF-κB(p65) or in combination with miR-99b agomir were detected by HE staining. D: W/D ratio in lung tissues of ALI mice treated with BAY11-7082, sh-NF-κB(p65) or in combination with miR-99b agomir. E: MPO activity in lung tissues of ALI mice treateSd with BAY11-7082, sh-NF-κB(p65) or in combination with miR-99b agomir. F: Inflammatory factor TNFα, IL-6, and IL-1β expression in the BALF of ALI mice treated with BAY11-7082, sh-NF-κB(p65) or in combination with miR-99b agomir was detected by ELISA. The measurement data were presented as mean ± standard deviation. The data of two groups were analyzed by independent sample *t test*. The one-way ANOVA was used in the comparison between multiple groups with Tukey's post-hoc test. * indicated *p* < 0.05, *n* = 8.

RT-qPCR findings showed that, compared with the ALI + DMSO group, the expression levels of miR-99b were inhibited and those of PRDM1 were enhanced in the ALI + BAY11-7082 group. Silencing of NF-κB(p65) led to an inhibited miR-99b expression with an elevated PRDM1 expression. Meanwhile, miR-99b expression levels were promoted, while those of PRDM1 were reduced in the ALI + sh-NF-κB(p65) + miR-99b agomir group compared with the ALI + sh-NF-κB(p65) + agomir NC group (Fig. 6*A*). Western-blot findings were consistent with RT-qPCR results (Fig. 6*B*).

In addition, HE staining illustrated an obvious decrease in the infiltration of inflammatory cells, edema of lung tissues (Fig. 6*C*), ratio of lung W/D (Fig. 6*D*), MPO activity in the BALF (Fig. 6*E*) and the secretion of pro-inflammatory factors in the lung tissues (Fig. 6*F*) of the ALI + BAY11-7082 group compared with the ALI + DMSO group. ALI + sh-NF-κB (p65) treatment brought about consistent results with those of the ALI + BAY11-7082 treatment. Meanwhile, the infiltration of inflammatory cells in lung tissues was increased (Fig. 6*C*), the ratio of lung W/D was elevated (Fig. 6*D*), MPO activity in BALF was elevated (Fig. 6*E*), and the concentration of inflammatory factor TNFα, IL-6, and IL-1β in lung tissues were all markedly increased (Fig. 6*F*) in the ALI + sh-NF-κB(p65) + miR-99b agomir group in contrast to the ALI + sh-NF-κB(p65) + agomir NC group. Collectively, these findings indicate that NF-κB(p65) promoted the progression of ALI in mice *via* miR-99b up-regulation to inhibit the PRDM1.

## Discussion

So far, ALI still presents with enormous fatality and morbidity rates (24). In this work, we demonstrated NF-κB(p65) promotion of miR-99b can affects the processes of ALI in LPS-induced MH-S cells and a murine model of LPS-induced ALI by down-regulating the expression of PRDM1.

Initially, our findings indicated that miR-99b was highly expressed in the LPS-induced mouse ALI models. Similarly, up-regulated levels of miR-99a have been previously identified in rat models of acute respiratory distress syndrome (25). We found that lung injury was increased in the ALI mouse models as evidenced by up-regulated W/D ratio, MPO activity and the concentration of TNFα, IL-6, and IL-1β, while the infiltration of inflammatory cells was reduced in the lung tissues, whereas all the aforementioned could be countered by the addition of miR-99b antagomir, which is very much in line with the previous data. Moreover, it has been documented that over-expression of miR-99b-5p brings about elevations in the expression levels of proinflammatory cytokines (IL-2, IL-6, TNFα, and IFN-γ), thus promoting the pathogenesis of rheumatoid arthritis (26).

Additionally, we uncovered the targeting relationship between miR-99b and PRDM1, and found that miR-99b interacted with the 3'UTR of PRDM1 mRNA, consequently inhibiting its expression. Indeed, miRNAs possess the ability to inhibit mRNA degradation or translation by interacting with the 3'UTR of specific target mRNAs (27). More in line with our findings, miR-125a (the miR-125a cluster on chromosome 19 in humans includes miR-99b) has been previously predicted to be able to bind to PRDM1 (28). Moreover, down-regulated levels of PRDM1 have been found in lung cancer cells, wherein these decreased expressions promoted cell invasion *in vitro* and lung metastasis *in vivo* (29). Furthermore, over-expression of PRDM1 is known to suppress the release of IFN-γ, TNFα, and TNF-β by direct binding to multiple conserved regulatory regions in human natural killer cells (30). As a result, we concur that miR-99b targeted PRDM1 expression to augment the state of LPS-induced cell injury.

ALI is characterized by inflammatory cell infiltration, pro-inflammatory cytokine generation (3, 31), along with ROS generation in the lungs (32). It has been reported that LPS treatment leads to ROS production and NF-κB activation (33), whereas these results hold true of our experimentation with MH-S cell models of ALI, wherein the expression levels of NF-κB (p-p65) in the nucleus were elevated, These aberrant levels of NF-κB(p-p65) were accompanied by increases in LPS concentration and NF-κB (p-p65) nucleation, while enrichment in the miR-99b promoter region was promoted by LPS treatment, which indicted that NF-κB (p65) was recruited to the miR-99b promoter region to promote inflammatory damage in its transcriptional regulatory cells. Existing data further reveals that NF-κB could promote the transcription of miR-99a by binding to the -1643 to -1652 region of the miR-99a promoter (34). In addition, one study suggested that NF-κB might be negatively-correlated with PRDM1 during the process of B-cell differentiation (35). These results collectively highlight the promoting effect of NF-κB on ALI via miR-99b-mediated PRDM1 inhibition.

**Figure 7:**
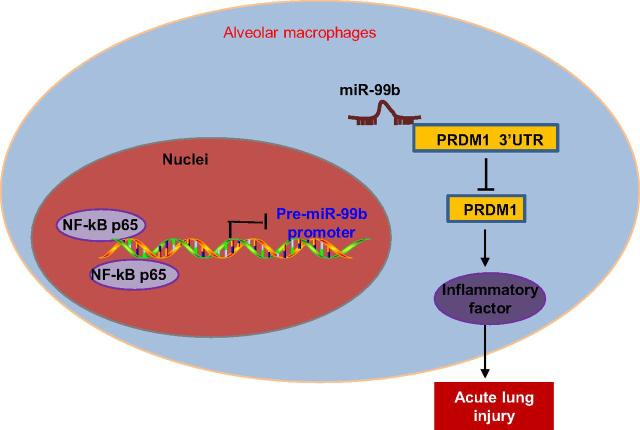
NF-κB(p65) elevates miR-99b expression by enriching in the miR-99b promoter region, causing the down-regulation of the miR-99b target PRDM1, thus promoting the progression of ALI.

In conclusion, the current study revealed that NF-κB(p65) promoted the miR-99b expression by enriching the miR-99b promoter region. miR-99b was highly-expressed in ALI mouse lung tissues and targeted PRDM1, causing its down-regulation, ultimately accelerating the progression of ALI (Fig. 7). Our findings suggesting that miR-99b might be important in changing the functions of PRDM1 in ALI. Therefore, miR-99b could serve as a therapeutic target for ALI according to our preliminary findings and warrants further investigation to fully-realize the importance of miR-99b.

## Materials and methods

### Establishment of mouse model of ALI

All animal experiments were approved by the Animal Care and Use Committee of Cangzhou Central Hospital. A total of 107 8–10-week adult male C57BL/6N mice were randomly grouped as the control (normal mice injected intraperitoneally with 30 mg/kg normal saline), ALI (mice injected intraperitoneally with 30 mg/kg LPS [L3129, Sigma-Aldrich Chemical Company, St Louis, MO, USA), ALI + antagomir NC (ALI modeled mice injected with miR-99b antagomir NC), ALI + miR-99b antagomir (ALI modeled mice treated with 50 g/L miR-99b antagomir), ALI + overexpression PRDM1-NC (oe-NC; ALI modeled mice treated with lentivirus expressing oe-NC), ALI + oe-PRDM1 (ALI modeled mice treated with 1 × 10^9^ pfu lentivirus expressing oe-PRDM1), ALI + sh-NC (ALI modeled mice treated with 1 × 10^9^ pfu lentivirus expressing sh-NC), ALI + short hairpin RNA targeting NF-κB (sh-NF-κB) (p65) (ALI modeled mice treated with 1 × 10^9^ pfu lentivirus expressing sh-NF-κB[p65]), ALI + BAY11-7082 (ALI modeled mice treated with 1 mg/kg of BAY11-7082, which is a NF-κB inhibitor), ALI + BAY11-7082 + agomir NC (ALI modeled mice treated with both 1 mg/kg of BAY11-7082 and lentivirus expressing agomir NC), and ALI + BAY11-7082 + miR-99b agomir (ALI modeled mice treated with both 1 mg/kg of BAY11-7082 and lentivirus expressing miR-99b agomir) (*n* = 8). As a result, 81 mice were successfully modeled, with a modeling success rate of 81.82%. Next, the mice were treated once by dropping 5 mg/kg LPS (using saline as a control) in the trachea after inducing anesthesia with 20 mg/kg pentobarbital sodium. BALF and lung tissues were subsequently collected 6 h after 5 mg/kg LPS infusion.

### Cell culture and grouping

MH-S cell lines and HEK293T cells were purchased from the cell bank of Chinese Academy of Sciences (Shanghai, China). Cells were cultured in Dulbecco's modified Eagle's medium (DMEM) supplemented with 10% fetal bovine serum (FBS) (Gibco, USA),100 IU/ml penicillin and 100 μg/ml streptomycin under 37 °C and 5% CO_2_. Meanwhile, the HEK293T cells were cultured in DMEM supplemented with 10% FBS in a 5% CO_2_ incubator at 37 °C, and then both were subjected to LPS treatment.

MH-S cells (4 × 10^5^ cells/well) at the logarithmic phase of growth were inoculated in a 6-well cell culture plate. The plasmids were connected to the PLV-Neo vector (Inovogen, China). The sequences were provided by Sigma Aldrich. Following sequencing and identification, the plasmids and PLV-Neo were co-transfected into the HEK293T cells. The groups were as follows: blank (PBS [PBS] treatment), LPS (10 μg/ml), LPS + inhibitor NC, LPS + miR-99b inhibitor, LPS + NF-κB inhibitor (10 μm BAY11-7082), LPS + NF-κB inhibitor (100 μm SATM), LPS + sh-NC, LPS + sh-NF-κB(p65), LPS + sh-NF-κB(p65) + miR-99b mimic, mimic NC, miR-99b mimic, oe-NC, oe-PRDM1, LPS + oe-NC and LPS + oe-PRDM1.

### HE staining

Deparaffinized mouse left lung tissues from different groups were made into sections (4-μm-thick). Next, the sections underwent staining for 7 min with hematoxylin and 1 min with eosin. Finally, histomorphology changes in the mouse lung tissues were observed under an optical microscope.

### Immunohistochemistry

Deparaffinized mouse left lung tissues from different groups were made into sections (4-μm-thick) and underwent immunohistochemical staining. The HistostainTMSP-9000 immunohistochemical staining kit (solarbio, China) was applied for staining. The primary antibodies against NF-κB(p65) rabbit antibody (ab16502, 1:500, Abcam Inc., Cambridge, UK) and PRDM1 rabbit antibody (ab106410, 1:200, Abcam Inc.) were added to the sections and probed at 4 °C overnight. After rinsing with PBS, the sections were re-probed with the secondary antibody. Next, the sections were colored with 3,3′-diaminobenzidine tetrahydrochloride (DAB) for 5–10 min, counterstained with hematoxylin for 1 min, sealed, and finally photographed. Positive criteria were as follows: five representative high-fold visual fields (positive optical microscope, Nikon, Tokyo, Japan) were selected for observation and counting, and the cytoplasm exhibited brown or yellow coloration.

### Changes in dry-wet proportion of lung

The mouse left lung surfaces were collected from different groups. The wet-weight of lung tissues was measured and recorded using an electronic scale. The weighed tissues were placed in an oven at 80 °C for 48 h, until there were no more changes in the weight. The dry weight of lung tissues was then measured and recorded, and the W/D proportion was obtained.

### MPO activity

Thiobarbituric acid (TBA) was added to lung tissue homogenates. The mixture was subsequently centrifuged, and the supernatant was determined using spectrophotometry to evaluate MPO activity. MPO activity of lung tissues was calculated as the absorbance change per gram.

### Immunofluorescence staining

The expression patterns of the CD68 protein were detected in the lung tissues using the immunofluorescence staining kit (Beyotime Biotechnology Co., Shanghai, China). In brief, the cells were fixed with 4% paraformaldehyde, treated with 0.1% Triton X-100 and washed twice with the immunostaining washing solution on a shaking table, 5 min each time, followed by the addition of immunostaining blocking solution to block the cells for 60 min. Thereafter, the cells were immunostained with the CD68 primary antibody (Cell Signaling Technology, USA) overnight at 4 °C. The following day, the FITC-labeled secondary antibody goat anti-rabbit (ab6717, 1: 1000, Abcam Inc.) was added to the cells and incubated at room temperature for 1 h. Subsequently, 4′,6-diamidino-2-phenylindole (DAPI) staining was performed on the nuclei for 5 min, and the slide was sealed, after which the expression patterns of the proteins were observed under a laser scanning confocal microscope.

### RT-qPCR

Total RNA content was extracted from the cells and tissue samples with the help of TRIzol reagents (Invitrogen Inc., Carlsbad, CA, USA). The RNA mass and concentration were detected using UV-vis spectrophotometry (ND-1000, Nanodrop Technologies Inc., Wilmington, USA). For miRNA, complementary DNA (cDNA) was obtained using miRNA First Strand cDNA Synthesis (Tailing Reaction) kits (B532453-0020, Sangon Biotech, Shanghai, China), and for mRNA, cDNA was obtained with reverse transcription kits (RR047A, Takara Bio Inc., Shiga, Japan). The fluorescence quantitative PCR was subsequently performed using cDNA as a template with reference to SYBR® Premix Ex Taq^TM^ II (perfect real time) kit (DRR081, Takara) instructions. The RT-qPCR reaction was carried out using a real-time fluorescence quantitative PCR instrument (ABI 7500, Applied Biosystems, Foster City, CA, USA) instrument. U6 and GAPDH mRNA levels were normalized as the internal parameters for the results. The primers are shown in Table 1, and 2-ΔΔCt represents the doubling relationship between the target gene expression of the experimental group and the control group.

**Table 1:** Primer sequences used for RT-qPCR

Targets	Forward (5‘→3‘)	Reverse (5‘→3‘)
miR-99b	CACCCGTAGAACCGACCTTGCG	
PRDM1	GGCTCCACTACCCTTATCCTG	TGCAGAGGTGCACATAGTCTG
P65	CGGGATGGCTACTATGAGGCTGAC	GATTCGCTGGCTAATGGCTTGCT
U6	CTCGCTTCGGCAGCACATATACT	ACGCTTCACGAATTTGCGTGTC
GAPDH	AGCAGTCCCGTACACTGGCAAAC	TCTGTGGTGATGTAAATGTCCTCT

### Western-blot

Total protein content was extracted from the cells or tissue samples using a radioimmunoprecipitation assay (RIPA) lysis buffer (C0481, Sigma-Aldrich, USA). The obtained proteins were separated with the help of PAGE, transferred to a PVDF membrane. Subsequently, the primary antibodies NF-κB(p-p65) rabbit antibody (ab86299,1: 2000, Abcam Inc.), NF-κB(p65) rabbit antibody (ab16502, 1: 1000, Abcam Inc.), and PRDM1 rabbit antibody (ab106410, 1: 1000, Abcam Inc.) were added to the membrane and incubated overnight. The following day, the membrane was washed thrice with TBST, 5 min each time, incubated with the HRP labeled goat anti-rabbit IgG (ab205718, 1: 20000, Abcam Inc.) for 1.5 h at room temperature, and added with the developer (NCI4106, Pierce, Rockford, IL, USA). GAPDH was used for protein quantitative analysis.

### CCK8 cell viability assay

MH-S cells were added with LPS (0.1, 0.5, 1, 5, and 10 μg/ml) for 24 h. Prior to LPS treatment, 10 μm BAY11-7082 and 100 μm SATM were added to the MH-S cells. Next, CCK-8 solution (Dojindo Laboratories, Kumamoto, Japan) with a volume of 10 μl was then added to each well and incubated for 2 h. Absorbance of each well was measured at 450 nm using an automatic porous spectrophotometer.

### Elisa

Mouse lung tissues (100 mg) were homogenized, centrifuged and the supernatant was collected. Additionally, mouse macrophage culture supernatant was collected. The levels of IL-1β, IL-6, and TNFα in the supernatant were determined using an avidin-biotin complex ELISA according to the manufacturing instructions. The ELISA kit was purchased from Xitang Biotechnology Co., Ltd. (Shanghai, China).

### ChIP assay

As per the instructions of the ChIP kit (Thermo Fisher Scientific Inc., Waltham, MA, USA), the treated cells were fixed with 1% formaldehyde and sectioned by ultrasonic treatment. Next, the NF-κB(p65) antibody (ab19870, dilution ratio of 1: 200, Abcam Inc., Cambridge, UK) was used for immunoprecipitation of p65-DNA complex. Afterward, the complex was filtered from DNA fragment with protein G agarose beads. The cross-linking of p65-DNA complex was reversed and the DNA chain was purified. RT-qPCR was then performed to quantify ChIP product. The primers of miR-99b promoter were as follows: Forward: 5′-GGTTGGGAAGGAGGGAAAGG-3′, and Re-verse: 5′-GAACTGGTCTTCTGGGGCTC-3′.

### Dual-luciferase reporter gene assay

3'-UTR plasmids of PRDM1 mutations and WT-PRDM1 containing the predicted binding site were constructed into psiCHECK vector. Macrophages were cultured in a 24-well plate with 400 ng firefly luciferase reporter gene plasmids and 25 ng Renilla luciferase constructed (pRL-SV40) in combination with 30 nm miR-99b mimic or mimic NC transfection reagents (RiboBio Co., Ltd., Guangzhou, china) according to the manufacturer's instructions. After 48 h of transfection, the activity of renilla luciferase and firefly luciferase was measured using a dual-luciferase reporter kit (Promega, USA). Internal firefly luciferase activity was standardized by renilla luciferase activity.

### Statistical analysis

Statistical analyses were performed using the SPSS 21.0 statistical software. Measurement data were presented as mean ± standard deviation. Comparison between two groups was conducted using an independent sample *t test*, and comparisons among multiple groups were based on one-way analysis of variance (ANOVA), followed by Tukey's post-hoc tests with corrections for multiple comparisons. A value *p* < 0.05 was considered statistically significant.

## Data availability

The datasets generated and/or analyzed during the current study are available from the corresponding author on reasonable request.

## References

[1] Rubenfeld G.D., Caldwell E., Peabody E., Weaver J., Martin D.P., Neff M., Stern E.J., Hudson L.D. (2005). Incidence and outcomes of acute lung injury. N. Engl. J. Med.

[2] Lemos-Filho L.B., Mikkelsen M.E., Martin G.S., Dabbagh O., Adesanya A., Gentile N., Esper A., Gajic O., Gong M.N., Illness U.S.C., Injury Trials Group: Lung Injury Prevention Study, I (2013). Sex, race, and the development of acute lung injury. Chest.

[3] Grommes J., Soehnlein O. (2011). Contribution of neutrophils to acute lung injury. Mol. Med.

[4] Bosmann M., Grailer J.J., Russkamp N.F., Ruemmler R., Zetoune F.S., Sarma J.V., Ward P.A. (2013). CD11c+ alveolar macrophages are a source of IL-23 during lipopolysaccharide-induced acute lung injury. Shock.

[5] Park J.R., Lee H., Kim S.I., Yang S.R. (2016). The tri-peptide GHK-Cu complex ameliorates lipopolysaccharide-induced acute lung injury in mice. Oncotarget.

[6] Zeng M., Sang W., Chen S., Chen R., Zhang H., Xue F., Li Z., Liu Y., Gong Y., Zhang H., Kong X. (2017). 4-PBA inhibits LPS-induced inflammation through regulating ER stress and autophagy in acute lung injury models. Toxicol. Lett.

[7] Luo M., Hu L., Li D., Wang Y., He Y., Zhu L., Ren W. (2017). MD-2 regulates LPS-induced NLRP3 inflammasome activation and IL-1beta secretion by a MyD88/NF-kappaB-dependent pathway in alveolar macrophages cell line. Mol. Immunol.

[8] Wang K., Zhu X., Zhang K., Yao Y., Zhuang M., Tan C., Zhou F., Zhu L. (2017). Puerarin inhibits amyloid beta-induced NLRP3 inflammasome activation in retinal pigment epithelial cells via suppressing ROS-dependent oxidative and endoplasmic reticulum stresses. Exp. Cell Res.

[9] Kailasan Vanaja S., Rathinam V.A., Atianand M.K., Kalantari P., Skehan B., Fitzgerald K.A., Leong J.M. (2014). Bacterial RNA:DNA hybrids are activators of the NLRP3 inflammasome. Proc. Natl. Acad. Sci. U S A.

[10] Rathinam V.A., Vanaja S.K., Waggoner L., Sokolovska A., Becker C., Stuart L.M., Leong J.M., Fitzgerald K.A. (2012). TRIF licenses caspase-11-dependent NLRP3 inflammasome activation by gram-negative bacteria. Cell.

[11] Willingham S.B., Allen I.C., Bergstralh D.T., Brickey W.J., Huang M.T., Taxman D.J., Duncan J.A., Ting J.P. (2009). NLRP3 (NALP3, Cryopyrin) facilitates in vivo caspase-1 activation, necrosis, and HMGB1 release via inflammasome-dependent and -independent pathways. J. Immunol.

[12] Davis B.K., Wen H., Ting J.P. (2011). The inflammasome NLRs in immunity, inflammation, and associated diseases. Annu. Rev. Immunol.

[13] Bauer C., Duewell P., Mayer C., Lehr H.A., Fitzgerald K.A., Dauer M., Tschopp J., Endres S., Latz E., Schnurr M. (2010). Colitis induced in mice with dextran sulfate sodium (DSS) is mediated by the NLRP3 inflammasome. Gut.

[14] Yekta S., Shih I.H., Bartel D.P. (2004). MicroRNA-directed cleavage of HOXB8 mRNA. Science.

[15] Trabucchi M., Briata P., Filipowicz W., Rosenfeld M.G., Ramos A., Gherzi R. (2009). How to control miRNA maturation?. RNA Biol.

[16] Newman M.A., Hammond S.M. (2010). Emerging paradigms of regulated microRNA processing. Genes Dev.

[17] Guo Z., Gu Y., Wang C., Zhang J., Shan S., Gu X., Wang K., Han Y., Ren T. (2014). Enforced expression of miR-125b attenuates LPS-induced acute lung injury. Immunol. Lett.

[18] Bjornsson K. (1994). Sygeplejersken. Confidentiality–Conscience.

[19] Ganan-Gomez I., Wei Y., Yang H., Pierce S., Bueso-Ramos C., Calin G., Boyano A.M.C., Garcia-Manero G. (2014). Overexpression of miR-125a in myelodysplastic syndrome CD34+ cells modulates NF-kappaB activation and enhances erythroid differentiation arrest. PLoS ONE.

[20] Napetschnig J., Wu H. (2013). Molecular basis of NF-kappaB signaling. Annu. Rev. Biophys.

[21] Romagnoli M., Belguise K., Yu Z., Wang X., Landesman-Bollag E., Seldin D.C., Chalbos D., Barille-Nion S., Jezequel P., Seldin M.L., Sonenshein G.E. (2012). Epithelial-to-mesenchymal transition induced by TGF-beta1 is mediated by Blimp-1-dependent repression of BMP-5. Cancer Res.

[22] Elias S., Robertson E.J., Bikoff E.K., Mould A.W. (2018). Blimp-1/PRDM1 is a critical regulator of Type III Interferon responses in mammary epithelial cells. Sci. Rep.

[23] Shen E., Rabe H., Luo L., Wang L., Wang Q., Yin J., Yang X., Liu W., Sido J.M., Nakagawa H., Ao L., Kim H.J., Cantor H., Leavenworth J.W. (2019). Control of Germinal Center Localization and Lineage Stability of Follicular Regulatory T Cells by the Blimp1 Transcription Factor. Cell Rep.

[24] Cross L.J., Matthay M.A. (2011). Biomarkers in acute lung injury: insights into the pathogenesis of acute lung injury. Crit. Care Clin.

[25] Huang C., Xiao X., Chintagari N.R., Breshears M., Wang Y., Liu L. (2014). MicroRNA and mRNA expression profiling in rat acute respiratory distress syndrome. BMC Med. Genomics.

[26] Zhu X., Wu L., Mo X., Xia W., Guo Y., Wang M., Zeng K., Wu J., Qiu Y., Lin X., Lu X., Deng F., Lei S. (2020). Identification of PBMC-expressed miRNAs for rheumatoid arthritis. Epigenetics.

[27] Ivey K.N., Srivastava D. (2015). microRNAs as Developmental Regulators. Cold Spring Harb. Perspect. Biol.

[28] Gururajan M., Haga C.L., Das S., Leu C.M., Hodson D., Josson S., Turner M., Cooper M.D. (2010). MicroRNA 125b inhibition of B cell differentiation in germinal centers. Int. Immunol.

[29] Zhu Z., Wang H., Wei Y., Meng F., Liu Z., Zhang Z. (2017). Downregulation of PRDM1 promotes cellular invasion and lung cancer metastasis. Tumour Biol.

[30] Smith M.A., Maurin M., Cho H.I., Becknell B., Freud A.G., Yu J., Wei S., Djeu J., Celis E., Caligiuri M.A., Wright K.L. (2010). PRDM1/Blimp-1 controls effector cytokine production in human NK cells. J. Immunol.

[31] Matute-Bello G., Frevert C.W., Martin T.R. (2008). Animal models of acute lung injury. Am. J. Physiol. Lung Cell Mol Physiol.

[32] Chung I.S., Kim J.A., Kim J.A., Choi H.S., Lee J.J., Yang M., Ahn H.J., Lee S.M. (2013). Reactive oxygen species by isoflurane mediates inhibition of nuclear factor kappaB activation in lipopolysaccharide-induced acute inflammation of the lung. Anesth. Analg.

[33] Cho R.L., Yang C.C., Lee I.T., Lin C.C., Chi P.L., Hsiao L.D., Yang C.M. (2016). Lipopolysaccharide induces ICAM-1 expression via a c-Src/NADPH oxidase/ROS-dependent NF-kappaB pathway in human pulmonary alveolar epithelial cells. Am. J. Physiol. Lung Cell Mol Physiol.

[34] Bao M.H., Li J.M., Luo H.Q., Tang L., Lv Q.L., Li G.Y., Zhou H.H. (2016). NF-kappaB-regulated miR-99a modulates endothelial cell inflammation. Mediators Inflamm.

[35] Piccaluga P.P., Agostinelli C., Fuligni F., Righi S., Tripodo C., Re M.C., Clo A., Miserocchi A., Morini S., Gariglio M., Ferri G.G., Rinaldi-Ceroni A., Piccin O., De Andrea M., Pileri S.A. (2015). IFI16 expression is related to selected transcription factors during B-cell differentiation. J. Immunol. Res.

